# Perianesthetic death in dogs and cats: a scoping review

**DOI:** 10.1177/10406387261421223

**Published:** 2026-02-17

**Authors:** Nicole Rose, Bruce Wobeser, Daniel J. Pang

**Affiliations:** Department of Veterinary Pathology, Western College of Veterinary Medicine, University of Saskatchewan, Saskatoon, Saskatchewan, Canada; Department of Veterinary Pathology, Western College of Veterinary Medicine, University of Saskatchewan, Saskatoon, Saskatchewan, Canada; Department of Veterinary Clinical and Diagnostic Sciences, University of Calgary Faculty of Veterinary Medicine, Calgary, Alberta, Canada

**Keywords:** anesthesia, autopsy, cats, death, dogs, pathology, postmortem examination, review, risk factors, veterinary

## Abstract

Perianesthetic death (PAD) is an uncommon yet devastating outcome in veterinary medicine, with incidence rates consistently higher than those reported in human anesthesia. In our scoping review, we summarize the current literature on PAD in dogs and cats and discuss definitions, risk factors, species-specific challenges, and the role of postmortem examinations. Although advances in veterinary anesthesia have occurred, inconsistencies in PAD definitions and follow-up periods have limited the ability to compare PAD incidence over time. Risk factors consistently associated with PAD include high American Society of Anesthesiologists physical status classification, age and weight extremes, species-specific traits, and procedural urgency. PAD risk is consistently higher in cats than dogs. Most studies rely on identifying clinical risk factors, with limited use of postmortem examination. When autopsies are used, they can reveal undiagnosed lesions that may have contributed to the animal’s death. However, many PAD autopsy cases yield no identifiable lesions, complicating determination of cause of death. Additionally, the lack of standardized submission forms and autopsy protocols further hampers consistency in case evaluation. We highlight the need for a multidisciplinary, standardized approach to the investigation of PAD cases. Future prospective studies should incorporate structured autopsy protocols and anesthesiologist reviews to improve diagnostic yield, with the goal of improving patient safety in veterinary anesthesia.

Perianesthetic death (**PAD**) in veterinary medicine is an infrequent but devastating complication. Drugs used in all phases of anesthesia alter many physiologic processes, affecting variables such as blood pressure, heart rate, and body temperature.^27^ Failure to recognize and address significant changes in these variables can have fatal consequences. Since the earliest veterinary PAD studies, many aspects of veterinary anesthesia have improved, resulting in an apparent overall decrease in the incidence of PAD. For instance, the incidence of PAD in dogs in the early 1990s was reported as ~0.23%; more recent work reports a reduction to 0.009%.^29,110^

In most studies, the rate of PAD in veterinary species is significantly higher than in humans. Human anesthesia studies report a PAD incidence of 0.001–0.01%.^59,69,72,119^ A study reporting a comparable PAD incidence of 0.009% in dogs was a single-center investigation conducted in a high-volume neuter clinic. The single-center and limited surgery focus is likely not representative of the general population. Perhaps a more realistic estimate of PAD in dogs is an incidence 10–100 times higher than that of humans, reflecting a clear need for further improvements in veterinary anesthesia.^59,69,72,119^

Variation in PAD incidence between human and veterinary anesthesia likely reflects differences in training, monitoring, and adhering to practice standards. For example, The American College of Veterinary Anesthesia and Analgesia (ACVAA) guidelines state that a responsible person must remain with the patient, although not necessarily focused solely on anesthesia.^10,29,36^ In contrast, human guidelines require constant monitoring by a physician or supervised anesthesia assistant.^34^ In addition to the different standards, another concern in veterinary medicine is inconsistent adherence to guidelines. For example, a 2020 study investigating anesthesia practice standards in English-speaking veterinary clinics in Québec Canada identified major differences in practice standards for managing veterinary anesthesia compared with published guidelines, particularly between referral practices and general practices.^116^ For example, blood pressure recording was performed in 55% of referral practices, but only 17% of general practices, despite ACVAA recommendations for measurement every 5 min.^10,116^ Given that patient monitoring is known to reduce the risk of perianesthetic death, the variation in practice monitoring standards may result in variation in risk of PAD as well.^19,38^

Veterinary studies have identified that patient and risk factors affect the incidence of PAD.^19,29,46 58, 110^ As a result, new practice standards can be implemented with the goal of reducing PAD.^82^ Patient risk factors identified consistently across studies are the American Society of Anesthesiologist (**ASA**) physical status classification, age, and weight. Anesthetic risk factors include the drug protocol used, urgency of the procedure, procedural duration and complexity, and the stage of anesthesia. The incidence of PAD also varies among species, with dogs having the lowest reported rate of the species for which data are available.

Interestingly, most veterinary PAD studies do not investigate lesions associated with death. If cause of death is reported, it usually is categorized into major systems, such as cardiovascular or respiratory.^19^ To date, only 4 PAD studies have described autopsy findings based on assessment by board-certified anatomic pathologists.^32,43,104,105^ We authored 2 of these papers, including a 2026 paper that, although published outside the February 2025 search criteria, we included here because of its relevance.^104,105^ These studies identified unexpected lesions that likely contributed to death and offered greater insight into underlying pathophysiology. However, a significant proportion (25–63%) of autopsy submissions across all 4 studies did not find significant lesions to explain the cause of death, resulting in an “unknown” cause of death.^32,43,104,105^ This highlights the need to improve investigation of PAD cases, which likely would involve obtaining a more detailed clinical history; standardizing autopsy protocol, including additional diagnostic modalities (e.g., toxicology); and consulting with other experts (e.g., anesthesiologists). In our 2026 article, we proposed a perianesthetic death postmortem checklist.^105^

Here, we summarize current information on PAD in veterinary medicine, with a focus on dogs and cats. We present the definition, incidence, and risk factors for PAD, followed by a discussion of the most common complications leading to PAD and the unique species-specific challenges associated with anesthesia. Finally, we review the benefits, challenges, and limitations of autopsy in PAD cases.

## Material and methods

We reviewed the literature to identify PAD studies in veterinary species. We searched the Web of Science, PubMed, and Scopus from the earliest date available until 2025 February 10. Search terms were “anesthesia or anesthetic or anaesthesia or anaesthetic” AND “death or mortality” AND “dog OR cat” AND NOT “human”, with our search limited to English-language articles. All search engines include variant spellings (e.g., results included the British English spelling for “anaesthesia”). Inclusion criteria were studies reporting clinical and/or autopsy findings in PAD cases in dogs and/or cats, and case reports of a single cat or dog with autopsy results. Exclusion criteria were studies reporting perianesthetic complications in a population undergoing a specific surgical procedure (e.g., complications associated with herniorrhaphy) or specific diagnostic procedures (e.g., magnetic resonance imaging of the head in cats), as these were either experimental anesthetic studies or case reports of very specific advanced procedures. We identified 633 studies in Web of Science, 939 in PubMed, and 579 in Scopus. Duplicates were removed, resulting in 575 Web of Science studies, 485 Scopus studies, and 854 PubMed studies. Study titles and abstracts then were screened to remove irrelevant articles. We reviewed the article reference lists to identify additional relevant studies, and included 10 additional studies. We authored one of the articles, which met our inclusion criteria,^104^ and we included another article that we published after the search date.^105^

## Definition, incidence, and risk factors for PAD

### Defining perianesthetic death

PAD definitions and research methods, including follow-up period and inclusion criteria, vary among studies, and therefore affect the reported PAD incidence. As a result, comparing the incidence of PAD over time and between studies is difficult.^21,37^

Criteria for determining what is considered an anesthetic procedure must be clear in PAD studies. A procedure may include general anesthesia, local anesthesia, and sedation.^24^ Some studies offer a very detailed definition for general anesthesia whereas others are either vague or do not provide a definition at all.^12,19,29,74,78, 96^ For example, in one the study, the definition was: “general anesthesia was considered to have been provided when an animal had been chemically restrained to the point of permitting endotracheal intubation and inhalation anesthetic had been administered including rare cases where a face mask was used.” Whereas in another study, the definition was: “chemical restraint sufficient to allow endotracheal intubation.”^19,74^ The difference in definition could affect which animals were included in different studies, resulting in fundamentally different study populations. Furthermore, some studies include sedation, which can alter physiologic function, in addition to general anesthesia.^19,68^

A second important component in defining PAD is the period from the end of anesthesia until death. In veterinary studies, this period varies considerably, including “until recovered,” “until discharge from the hospital,” 24 h, 48 h, 72 h, and 7 and 14 d.^6,17,19,42,44,54,55,56,57,73,74,109,110,120^ Occasionally, the time period is not described.^29,87^ A shorter time period minimizes loss of patients to follow-up and reduces the likelihood of including deaths unrelated to anesthesia.^19^ The Confidential Enquiry into Perioperative Small Animal Fatalities (**CEPSAF**) study investigated deaths up to 48 h after the end of anesthesia, and found that most deaths occurred within the first 3-h post anesthesia; therefore, these shorter time periods may be appropriate.^19^ A 2022 study examined 157,138 dog and cat anesthetic procedures at 2 different times—48 h and 2 wk post-anesthesia.^110^ PAD incidence increased from 0.10% (159 deaths) at 48 h to 0.14% (219 deaths) at 2 wk.^110^ The 60 additional deaths included euthanasia cases or death where anesthesia could not be excluded as a contributing factor.

Criteria used in determining whether death is related to anesthesia also vary. Early studies did not define which deaths were included, included only deaths resulting from cardiac arrest, or included all deaths regardless of cause.^36,38,55^ The CEPSAF study was the first large-scale small animal study to use an independent panel to review anesthetic records and assign a cause of death. This study excluded deaths from surgical or preexisting conditions, such as severe hemorrhage from a splenic tumor during surgery.^19^ Interestingly, the CEPSAF study reported that only 10% of the cases had an autopsy performed.^19^ Autopsies could help further clarify cause of death or generate a list of likely contributing factors.

In human medicine, PAD cases can be classified in 1 of 4 categories: 1) “death caused by the disease or injury for which the invasive procedure or anesthesia was being performed”; 2) “death caused by a disease or abnormality other than that for which the procedure was being performed”; 3) “death resulting from a mishap during, or a complication of, the invasive procedure”; 4) “death resulting from mishap during, or as a complication of, the administration of an anesthetic.”^62^ A pathology-focused study recommends using these categories when performing autopsies to investigate PAD.^32^ A suggested case definition for anesthetic death in veterinary medicine could, for example, be the following: death or euthanasia within 14 d of anesthesia or sedation for which anesthesia cannot be ruled out as the sole cause or contributing factor (based on clinical history and postmortem examination), after excluding underlying disease and/or surgical complications as the sole cause of death. Although postmortem examination cannot always determine with certainty how a case should be categorized, it may help rule in or out certain processes and serve as a tool in better understanding PAD.

### Perianesthetic death studies

Over the past several decades, advances in veterinary anesthesia have occurred through increased education, improved anesthetic procedures, and better patient stabilization and monitoring.^13,19,74^ Some improvements, such as patient stabilization and use of pulse oximetry, have reduced PAD.^13,28^ Consequently, the incidence of PAD would be expected to decrease over time. However, treatment options also have advanced over time, meaning that more animals with a higher ASA status are being anesthetized, potentially resulting in more PADs.^2^ An increased number of sick animals anesthetized was indicated by the CEPSAF study, in which sick cats (ASA status III–V) were 8% of all cats reported in the study. This contrasted with earlier work performed almost 20 y before CEPSAF, in which sick cats were 4% of 20,103 cats.^18,29^

Other factors shown to influence PAD incidence include study method (retrospective questionnaire vs. prospective) and clinic type (general vs. referral). Earlier studies were often based on retrospective questionnaires sent to veterinarians.^36,60,61^ Response rates were 18–45%, reflecting a potential for selection bias and a lack of generalizability.^36,60^ Additionally, earlier questionnaires relied heavily on reporter recall, potentially underestimating the true incidence of PAD.^35,36,60^ For instance, in one study, the questionnaire asked “How many animals do you believe have died as a result of anesthesia in the last two years?”^36^

Clinic type can also influence PAD incidence. Referral centers tend to have a higher incidence of PAD compared with general practices.^12,19,24,57^ Using dogs as an example, studies that focused on general practices only report PAD of 0.05–0.23%, whereas studies in referral or teaching practices report an incidence of 0.43–1.49%.^12,13,29,38,42,44,55,57,74,95^ Animals treated at referral practices are often sicker and undergoing more advanced/invasive procedures.^12^ This may be balanced by referral practices, which tend to follow recommended anesthesia practice guidelines and have access to advanced treatment options (e.g., blood transfusion).^65,116^ Differences in patient populations among clinics are supported by reports that <5% of general practices and >25% of referral practices anesthetize animals with an ASA status ≥III.^12,29^

Despite methodologic variations among PAD studies, research from the same institution, or from studies following the same criteria, facilitate comparison between studies. Results in dogs from the 2008 CEPSAF study reported a PAD incidence of 0.17% (95% CI [0.14, 0.19%]), and a 2022 study using the same study methodology by some of the same research team reported a PAD incidence of 0.10% (95% CI [0.09, 0.12%]).^19,110^ In another study, the researchers assessed risk of PAD at U.S. hospitals owned by the same corporation before and after implementing medical quality standards (MQS) through clinical audit.^82^ Before MQS implementation, the PAD rate in dogs and cats was 7.4 deaths per 10,000 procedures (0.074%).^82^ Six months after adopting MQS, the PAD rate declined to 6.2 deaths per 10,000 procedures (0.062%)—a statistically significant 16% reduction.^82^ The decrease in mortality resulted in an estimated survival benefit of more than 100 additional pets per year, with approximately 2,038,318 dog and 350,410 cat anesthetic episodes occurring over the course of the study.^82^

### Risk factors

Most PAD studies investigate clinical risk factors, with few studies describing lesions. By identifying major risk factors, recommendations can be made to reduce them. However, postmortem examination is rarely performed and thus risk factors may be poorly understood.^19^ Major risk factors associated with PAD are patient characteristics (e.g., ASA status) and anesthesia or surgical variables (e.g., level of monitoring, urgency of procedure).^11,111,112^ Study results vary regarding which factors are associated with an increased risk of PAD, likely reflecting the complex nature of the disease. However, information acquired from these studies has contributed to re-evaluating anesthesia standards.^19,74^

#### Risk factors associated with patient characteristics

Using the ASA classification system, a patient’s physical status can be categorized based on history and physical examination to help determine the patient’s associated risk for anesthetic complications (**Table 1**; **Suppl. Table 1**).^23,51,92^ A consistent finding across studies and species is the positive correlation between increasing ASA status and risk of PAD.^18,19,29,37,38,50,53,54,58,80,96,97^ As an example, in a systematic review of ASA status, dogs with an ASA status ≥III had 4.73 times the risk of PAD compared with dogs that had an ASA status <III.^92^ A similar trend was observed in cats.^92^

**Table 1.** Important clinical history to obtain for perianesthetic death autopsy submissions. Additional suggestions are reported in the right column.

Parameter	Gerdin et al.^43^	Suggested additional information
Patient information	Signalment, presenting complaint.	Signalment. Age, sex, neuter status, species, breed. If brachycephalic breed, severity of brachycephaly (e.g., stertor, dyspnea, exercise intolerance). Weight and body condition score.
Patient health	ASA classification, significant pre-anesthetic physical examination findings.	Ancillary test results (bloodwork, diagnostic imaging, etc.). Is animal up to date on vaccines and deworming? Any current illnesses and treatments (including medication and doses)? What was the animal’s American Society of Anesthesiologist physical status (ASA) classification on the day of the procedure, and why was this value chosen?
Day of procedure	Not reported.	Date of the procedure and date of death. Pre-anesthetic examination on day of procedure (TPR, temperament, etc.). Was the patient confirmed fasted and for how long?
Procedure	Purpose of anesthesia/sedation.	What procedure was being performed? Were there any complications during the procedure (e.g., excessive bleeding)?
Stages of anesthesia	All medication: drug, dose, route, time given. Premedication. Induction. Maintenance.	All drugs, dose, route, volume, and time given.
Anesthesia	Endotracheal intubation (Y/N).	Was the patient intubated? Were there any issues with intubation? If the animal was extubated, were there any issues with the extubation? How often was patient monitored? What parameters were monitored and how? Were any parameters abnormal during the procedure? If yes, were they corrected and how? Was fluid therapy provided? If so, how much did the patient receive and by what route? How long was the anesthesia and surgery?
Recovery	NA.	How was the recovery? What were the vital parameters on recovery? How often was the patient monitored on recovery?
Complication	Time arrest was noted. Type of arrest (respiratory or cardiopulmonary). Oxygen content inspired/delivered at time of death.	Time complication was noted. Were any procedures being performed around the time of the complication (e.g., injection, manipulation)? Were there any abnormalities leading up to the complication? Was the animal intubated at the time of the complication? How did the animal die (i.e., found dead, cardiac arrest, euthanized)? Did any vomiting occur throughout the perianesthetic period? Were there any non-fatal complications associated with the procedure? (e.g., hypotension)
Cardiopulmonary resuscitation (CPR)	Attempted (Y/N). CPR drugs/reversal given (dose and route). Oxygen content delivered. Duration of CPR.	Was CPR performed? CPR drugs/reversals given (dose, route, time). Was the animal intubated? Was oxygen being given? How long did the CPR last? What procedures were performed during CPR (e.g., chest compressions, intracardiac injections, open cardiac massage)?
After death	Body handling since time of death (refrigeration freezing, thawing, etc.).	
Specialty opinion	NA	Would you be willing to provide postmortem radiographs before submitting the body for review by a veterinary radiologist? Would you be willing to provide the anesthetic records for review by a veterinary anesthesiologist?
Additional information	NA	Do you have any listed differentials for the cause of death? Do you have any additional comments?

A challenge with assigning ASA status is interobserver variability.^77^ The ASA classification system has not been validated in either human or veterinary medicine (Suppl. Table 1).^51^ In veterinary medicine, detailed clinical history is not always available, and physical examination may be limited by temperament.^4^ As a result, preexisting health conditions may go unrecognized. This could lead to a lower ASA status being assigned, falsely decreasing the risk assessment. Interestingly, many PAD studies, including those that use ASA status, assign status retrospectively based on review of the anesthetic chart because ASA status was not assigned at the time of anesthesia. Retrospective assignment represents a potential source of ASA status inaccuracy.^29,66,74^ One study reported that only 46 of 131 (35%) respondents evaluated ASA status before routine surgeries (the study did not clarify what is considered a routine surgery).^116^ It is concerning that veterinarians are not regularly documenting ASA status before anesthesia despite its association with anesthetic risk.^20,29^

Increased age has been reported as a risk factor for PAD among dogs and cats.^54,55,74,96^ Cats ≥12-y-old are twice as likely to die under anesthesia than cats 0.5–5-y-old. Dogs ≥12-y-old are ~7 times more likely to die from anesthesia than dogs 0.5–8-y-old.^18,20^ Pediatric puppies (<3-mo-old) have a higher risk than adult dogs.^96^ Underlying reasons for the effects of age are likely multifactorial, reflecting age-related differences in physiology and likelihood of co-morbidities being present.^30,83^ Additionally, available anesthetic and monitoring equipment may not be suitable for different patient sizes and ages. Notably, not all studies have established a link between age and risk of PAD.^12,13^

Body weight is another characteristic influencing PAD risk. In dogs, low body weight (<5 kg) increased risk of PAD 8-fold compared with body weights of 5–15 kg.^20^ This association may reflect risk of hypothermia and drug overdosing attributed to poor body condition.^30,74,97^ Small body size might partially explain the higher risk of PAD in cats compared with dogs.^19^ Body weight extremes and obesity have been associated with increased PAD risk in cats and dogs, respectively.^20,74,96^ Overweight animals may receive higher doses of anesthetic agents if not dosed appropriately using lean body weight and may have increased respiratory compromise resulting from positional compression of the lungs by abdominal contents.^18^

#### Risk factors associated with anesthesia and surgery

Anesthetic and surgical factors—including patient monitoring, procedure type and urgency, anesthetic drugs, and stage of anesthesia—have been associated with risk of PAD. The clinical practice in veterinary medicine can vary significantly particularly when looking at the level of monitoring and personnel experience.^50,73^ Increased monitoring by a designated person, evaluating multiple parameters, results in a reduced risk of PAD.^19,74^ In human medicine, anesthesiologist experience has been associated with improved patient safety.^45^ These studies report that both level of monitoring and clinical experience play important roles in anesthesia safety, likely because early warning signs of instability are detected in the patient and changes are implemented before the problem escalates.^50,74^

Procedure type and urgency are linked to risk of PAD.^19,74^ Nonelective procedures increase PAD risk because these procedures are often performed in animals with more severe disease and/or on an emergency basis.^74,97^ Emergency procedures frequently occur outside of normal working hours when fewer staff may be available; fatigue may also play a role.^22^ Interestingly, in a 2023 study, non-urgent but unscheduled anesthesia was associated with a higher risk of PAD.^96^

The role of specific anesthetic agents in PAD has been evaluated in numerous veterinary studies.^29,38,117^ Drugs associated with an increased risk of PAD (e.g., xylazine) support use of newer alternatives (e.g., medetomidine).^29,38^ However, different studies report conflicting results. Use of acepromazine in both equine and small animal patients is associated with reduced risk of PAD in some studies, whereas a positive or negative association was not observed in other studies.^29,38^

Interestingly, mechanical ventilation has been associated with an increased risk of PAD in cats.^97^ The authors speculated this might reflect the challenges of mechanically ventilating smaller patients and the risk of barotrauma; also, cats requiring mechanical ventilation might have significant co-morbidities.^97^

**Figure 1.**
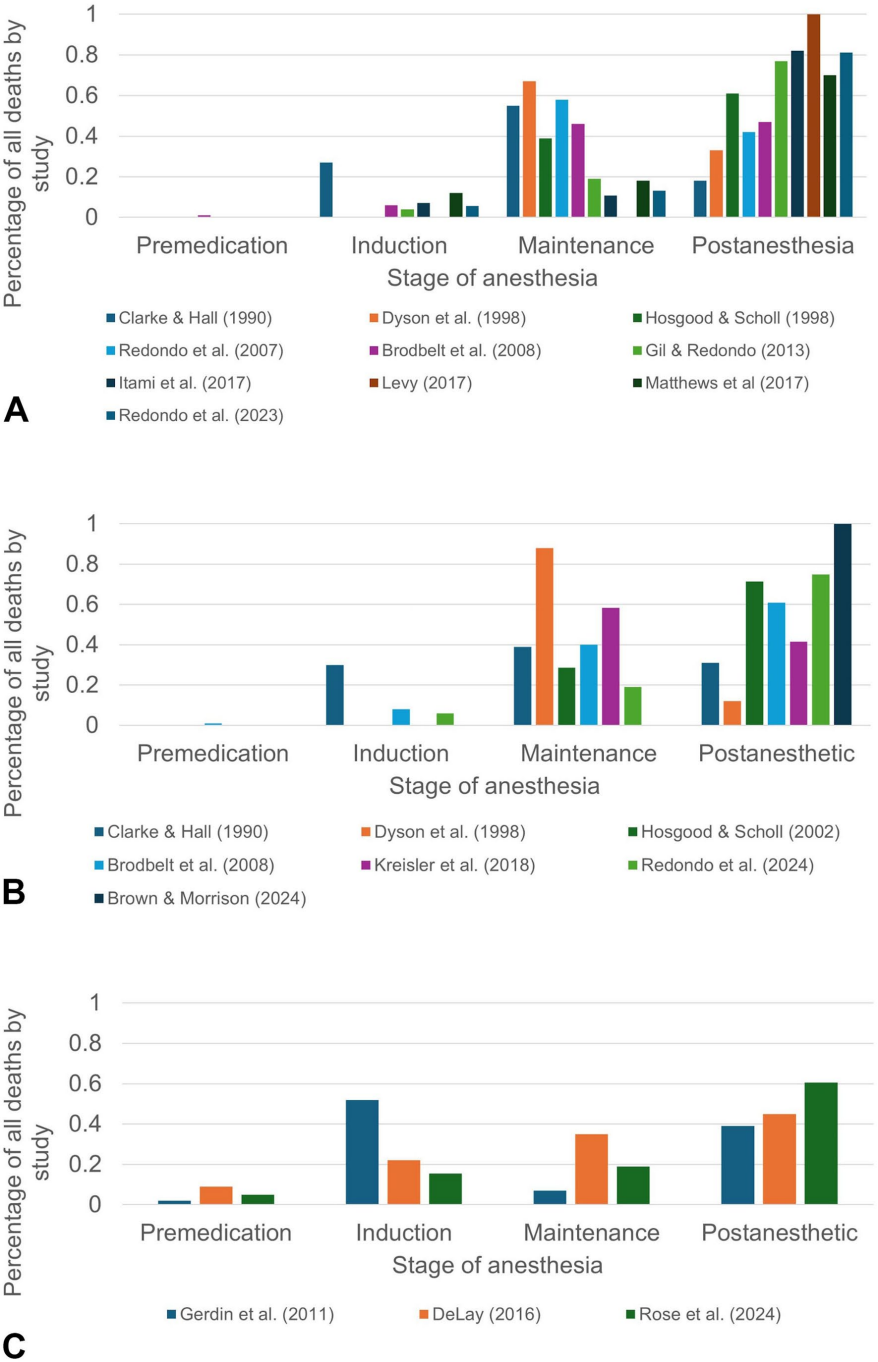
Stage of anesthesia where perianesthetic death occurred: **A**) dog studies; **B**) cat studies; **C**) postmortem studies. In Fig. 1C, Gerdin et al. cats only^43^, DeLay multiple species^32^, and Rose et al. 2024 dogs and cats.^104^ Induction = the induction agent is given, the animal is intubated, and full monitoring commences. Maintenance = the patient has an endotracheal tube in place and is receiving inhalant anesthesia. Postanesthetic = the animal has been extubated to the end of the observation period (time length varied between studies). Premedication = the animal received sedation in preparation for general anesthesia. Values were calculated as a percentage of all animals by study in which stage of death was reported.

Finally, the stage of anesthesia during which PAD occurs appears to have changed over time (**Fig. 1**).^21^ Anesthesia can be divided into 4 stages: premedication, induction, maintenance, and post-anesthesia. Earlier studies found induction and maintenance were high-risk periods for PAD. More recent studies have identified the first 3 h post anesthesia as the highest risk period.^22,53,68,97,110^ Contributing factors may include increased oxygen demand during recovery, risk of airway obstruction following endotracheal tube removal, and less monitoring equipment being used.^99^

### Species-specific differences

#### Dog anesthesia

Since the 1990s, the reported incidence of PAD in dogs has been 0.05–1.49% (Suppl. Table 2).^19,55^ Earlier studies tended to use vague definitions of PAD and calculate incidence based on respondent estimates, possibly underestimating the true incidence.^29,36,61^

Few PAD studies in dogs (<30%) attempted to categorize cause of death (Suppl. Table 2). When categorized, causes were often vague; for instance, terms such as “cardiac arrest” or “cardiovascular collapse” can represent several different etiologies.^20,29^ Further, authors seldom described how cause of death was determined and by whom. Only 3 clinical studies investigating canine PAD reported the use of postmortem examination (Suppl. Table 2); however, within these studies, <12% of cases performed a postmortem examination.^19,29,38^ Two studies reported the use of an independent panel of specialists to categorize cause of death.^19,57^ This approach allowed for a more detailed classification of the causes of PAD.^19^ However, given that categories were limited to major organ systems (e.g., cardiovascular, respiratory) and these studies still reported 21−25% of deaths as unknown, this approach leaves room for improvement.^19,57^ This proportion of unknown causes could reflect limited monitoring, undiagnosed underlying disease, limited use of autopsies, absence of standardized autopsy protocols, lesion-free complications, interpretive artifacts (e.g., cardiopulmonary resuscitation [**CPR**]), and poor body preservation.

Compared with cats, dogs have larger phenotypic variability; known breed-specific perianesthetic conditions have been summarized. ^40^ Brachycephalic breeds have been variably reported to have an increased risk of peri-anesthetic complications.^48,70^ They have many anatomic changes that increase the risk of upper airway obstruction.^81^ No standard definition exists as to what is classified as a brachycephalic breed, but tend to include Pug, English Bulldog, French Bulldog, Boston Terrier, Boxer, Shih Tzu, Pekingese, and Cavalier King Charles Spaniel.^71,87,89^ Changes directly associated with the respiratory tract include stenotic nares and an elongated soft palate.^39,87,91^ Secondary lesions associated with the increased negative pressure in the upper airway include nasopharyngeal edema, rhinitis and/or pharyngitis, everted laryngeal saccules, everted tonsils, and compromise of the laryngeal and tracheal cartilage. Other clinically relevant anatomic features in these breeds include tracheal hypoplasia and chronic gastroesophageal reflux that could increase the risk of traumatic endotracheal intubation or aspiration pneumonia, respectively.^39,87,91^ Although brachycephalic breeds are generally associated with an increased risk of anesthetic complications, including PAD, some studies did not identify a higher risk of developing anesthetic complications.^38,38,110^

#### Cat anesthesia

Over the past 40 y, the reported incidence of PAD in cats has been 0.06–0.43%, with one exceptionally high report of 5.1% (**Suppl. Table 3**). The incidence of PAD is generally higher in cats than in dogs.^52^ The most accurate PAD estimate is likely 0.1–0.3%, a range that reflects multi-institutional studies. Other studies were retrospective surveys that may have under-represented PAD incidence (<0.1%), single-center studies of teaching hospitals that reported higher incidence (>0.3%), or those that included euthanasia within the PAD definition, resulting in an inflated incidence (5.1%).

Five clinical studies reported cause of death (Suppl. Table 3); however, most were nonspecific causes.^19,29,38,42,54,120^ Attributing cause of death to cardiac arrest is nonspecific, given that many etiologies can lead to this outcome (see cause of death section). A discrepancy also exists between cause of death based on chart review and autopsy.^38,107,120^

Two studies identified an increased risk of PAD in cats with endotracheal intubation during anesthesia.^18,29^ In general, endotracheal intubation in cats tends to be more technically challenging than in dogs. Cats have a smaller, more sensitive airway, and stimulation from an endotracheal tube can lead to laryngospasm or edema, resulting in airway obstruction.^100^ Inexperience with intubation could explain the association of PAD with orotracheal intubation. Intubation was not routinely performed when older studies were conducted. A 1992 study reported that 74.5% of small animal practitioners did not routinely perform endotracheal intubation in cats.^36^ Alternatively, cats with tracheal intubation may have been at greater risk when undergoing longer or more invasive procedures, possibly confounding the association between intubation and PAD. Despite these historical associations, tracheal intubation in cats generally provides a secure airway and remains the standard of care during general anesthesia for all but the shortest procedures.^47,100^

Another surprising PAD risk factor identified in cats was the use of fluid therapy.^18^ Fluids are routinely used during anesthesia to offset ongoing losses, help maintain arterial blood pressure, and improve perfusion. However, cats may be particularly susceptible to inadvertent fluid overload because of their small body size or as a consequence of underlying cardiac disease.^27,32^ A PAD study reported that 21 of 90 (23%) cats submitted for autopsy had underlying cardiomyopathy, although only 4 were identified with clinical disease before anesthesia.^32^ Without regular postmortem examination on feline PAD cases, it is unknown if undiagnosed cardiac disorders are a significant risk factor. A standard autopsy that includes weighing the animal and comparing the pre-surgical weight and autopsy weight may allow the detection of more subtle fluid changes, with a 10% increase supportive of fluid overload.^114^

Rare causes of PAD reported in cats include suspected malignant hyperthermia and muscular dystrophy. ^98,115^

### Lesions associated with perianesthetic death

Only 4 studies reported autopsy findings identified by a board-certified anatomic pathologist in cases of PAD.^32,43,104,105^ The 2011 report was a retrospective study of PAD in cats at 2 neuter centers in New York State in 2009–2010.^43^ Cats anesthetized at these clinics were either from shelters, low-income households, unowned but with regular human contact, or feral. All cats were examined following a standard procedure and within 12–48 h of receiving the body. PAD was defined “as death within 24 h of receiving anesthetic agents,” with 54 cases meeting the inclusion criteria. The number of anesthetic procedures performed at the submitting clinics was not reported, precluding determination of anesthetic death rate.^43^

The 2016 autopsy study retrospectively identified PAD cases in a variety of species submitted to a university pathology laboratory from 2007–2015.^32^ PAD was defined as “death that occurred within 24 h of sedation or premedication for general anesthesia, or for sedation alone, and for which autopsy was carried out at the Animal Health Laboratory,” with 221 cases meeting the inclusion criteria (218 of 221 cases also had histology performed).

We performed the 2 other studies.^104,105^ In our 2024 study,^104^ we retrospectively investigated perianesthetic- and sedation-related deaths in dogs and cats submitted for autopsy to 4 diagnostic laboratories in Canada from 2012–2022. A total of 232 dogs and 193 cats met the inclusion criteria of whole-body submissions for postmortem examination after a perianesthetic or sedation death within 7 d of the procedure.^104^ In our 2026 study,^105^ we examined trends in feline perianesthetic deaths in a referral teaching hospital and general practices in Saskatchewan, Canada. Whole bodies of 45 cats from general practices and 23 cats from referral practices were submitted for postmortem examination after dying within 7 d of general anesthesia.^105^

All these studies have limitations, including insufficient descriptions of lesions, such as severity, and the criteria used by the pathologist to determine whether a lesion was a significant factor in death. For example, what diagnostic criteria for hypertrophic cardiomyopathy were applied, and which lesions specifically supported its role as cause of death? Despite their limitations, these studies provided valuable information to help further characterize cause of death in a number of cases and identified areas for improvement.

Few clinical PAD studies describe the significant autopsy lesions that contributed to the death of the animal (Suppl. Table 2). Of those studies, few had postmortem examinations performed by pathologists, with the CEPSAF study reporting the use of pathology reports in only 10% of the cases.^19^ The major reported PAD categories are cardiovascular, respiratory, other, or unknown. However, many studies do not describe the criteria for classification or use only vague descriptors.

#### Cardiovascular complications

Cardiovascular complications that result in inadequate oxygen delivery to vital tissues are often implicated in PAD.^27^ Cardiac arrest, a commonly reported cause of PAD, can be secondary to numerous, often multifactorial, etiologies, including hypoxemia, medication overdose, hypovolemia, severe electrolyte or acid-base disturbance, or preexisting cardiac disease.^27^ In small animal clinical studies, cardiovascular causes of PAD were recorded in 0–30% of cases; the most common reasons were cardiac arrest, cardiovascular collapse, hypovolemia, or hemorrhage.^19,27,57,95,110^ Two clinical PAD studies reported detailed lesions, including right ventricular dilated cardiomyopathy and dirofilariosis.^107,120^

Autopsy findings may alter the diagnosed cause of PAD. For example, in 1 study, 96% of cats were recorded as dying from cardiac arrest, yet only 11% had significant cardiac lesions, such as hypertrophic cardiomyopathy or myocarditis.^43^ However, the absence of structural lesions does not exclude cardiac arrest secondary to acute hypoxia, electrolyte imbalance, or drug toxicity, which are better assessed through serum chemistry, toxicology, or potentially cardiac-specific troponin measurement. The cardiac conduction system is extremely difficult to adequately evaluate at autopsy—structural abnormalities (such as atrial dilation) cannot be used to confirm functional disturbances (such as arrhythmia). Thus, although autopsy may help refine the cause of PAD, its limitations should also be recognized.

In the autopsy study investigating lesions across multiple species,^32^ 61 of 221 (28%) cases had significant cardiac lesions. Lesions were considered significant if the pathologist deemed them clinically relevant to the animal’s death. Cardiac lesions considered significant were found in 26 of 105 (25%) dog deaths and 29 of 90 (32%) cat deaths, with cardiomyopathy being the most identified lesion.^32^ None of the dogs and only 4 of the cats with cardiac lesions had a history of cardiac disease before anesthesia.^32^ Because the study was retrospective, it is unknown if cardiac lesions were associated with clinical signs or if the submitting clinician did not provide (or forgot to report) this information. However, because ~34% of dogs and 90% of cats with cardiovascular lesions were undergoing elective procedures, it is likely that signs of cardiac disease were not identified pre-operatively.^32^

In these autopsy PAD studies, despite identifying the heart as a significant contributor to PAD, only ~62% of cat hearts and 16% of dog hearts were weighed. Heart weight-to-body weight (HW:BW) ratio can support the diagnosis of cardiac disease and should be recorded as part of a standard PAD autopsy.^101^ Importantly, although reference values for HW:BW ratios exist for some species, interpretation may be confounded by extreme body condition. Interestingly, in laboratory animal medicine, the HW-to-tibial length ratio is used to identify cardiomegaly, given that tibial length remains constant after maturity and therefore is not susceptible to variation in BW.^122^ The application of this method in companion animals may be a future area of research; however, challenges might be encountered with breeds that have shortened limbs attributed to genetic skeletal differences (e.g., English Bulldogs).

#### Respiratory complications

Anesthesia alters respiratory function and physiology; it is therefore not surprising that respiratory disease is a common cause of PAD.^19,32,43,104,105^ In clinical PAD studies of dogs and cats, 10−50% of deaths were associated with the respiratory system.^19,29,38,57,120^ Listed respiratory causes of death were most frequently reported in cats and included laryngospasm, aspiration pneumonia, upper airway obstruction, and diaphragmatic hernia.^9,29,38,107,120^,

One of the most serious and rapidly fatal respiratory complications is airway obstruction. Numerous causes can lead to obstruction, including abnormal positioning of anatomic features during anesthesia (e.g., the soft palate contacting the tongue in brachycephalic breeds), edema of the upper respiratory tract, laryngospasm, tracheal collapse, accumulation of blood or mucus within the endotracheal tube, and/or kinking of the endotracheal tube.^9,25,27^ In an autopsy study of animals that died from respiratory lesions, ~32% of had lesions associated with obstruction of the airway, including upper respiratory tract obstruction (e.g., mucus plug), brachycephalic syndrome, tracheal collapse syndrome, laryngospasm, and laryngeal edema.^32^

Aspiration of gastric contents is another potentially fatal respiratory complication.^63^ In an autopsy study, the authors identified acute aspiration pneumonia as the most significant lesion contributing to PAD in 19% of respiratory tract lesions, including 5 dogs, 1 cat, and 1 horse.^32^ Other complications associated with the respiratory tract include atelectasis, space-occupying lesions in the lungs or pleura, tracheal tears, drug-induced hypoventilation, cardiogenic and non-cardiogenic pulmonary edema, pulmonary thromboembolism, and acute respiratory distress syndrome.^8,15^

Given the large number of potential respiratory complications, autopsy PAD studies are expected to identify significant and/or unexpected respiratory lesions interpreted by the pathologist as contributing to the animal’s death.^32,43,104,105^ In the neuter autopsy study, the authors identified underlying lung disease in 13 of 54 (24%) cats studied.^43^ Diseases included lungworm infection (*n* = 5), asthma (*n* = 3), acute aspiration (*n* = 1), and pneumonia (*n* = 4).^43^

**Figure 2.**
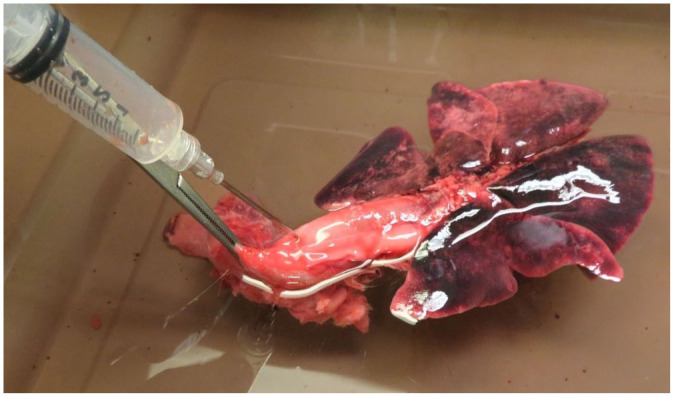
Pluck from a cat submerged in a bucket of water. The most cranial portion of the trachea has been clamped, and a 10-mL syringe filled with air penetrates the trachea and injects air. Any defects along the tract will result in air bubbles within the water.

Recommended autopsy assessment of the respiratory tract includes opening the glottis, evaluating the soft palate and the integrity of the tracheal mucosa, and documenting the presence of obstruction, disease in the pleura, and any evidence of lung disease.^32,105^ Furthermore, possible specific procedures, such as immersing the pluck in water, might aid in identifying lacerations along the respiratory tract (**Fig. 2**).^32,105^

#### Other complications

Causes of PAD unrelated to the cardiopulmonary system are less frequently reported in both clinical and autopsy studies. Complications in other organs may take a few days to weeks post-anesthesia to develop (e.g., ischemic injury in the kidney secondary to hypotension). These may go unrecognized as being associated with PAD, especially in studies that limit the follow-up period to immediately after surgery or to only a few days.^86^ Complications associated with the gastrointestinal tract can occur along any segment. The esophagus can be damaged if the animal regurgitates during anesthesia or if the animal has preexisting gastroesophageal reflux, a condition that is exacerbated by anesthesia.^121^ Sequelae of esophageal mucosal contact with acidic gastric contents can lead to esophagitis, stricture, or rupture.^1^ Causes of PAD associated with the urinary system include post-anesthetic renal failure and acute tubulointerstitial nephritis.^19,79^ Nervous system causes of PAD can be difficult to determine without additional testing, including blood glucose levels or postmortem examination, but uncontrolled seizures have been reported.^20^

In the multispecies autopsy study, the researchers identified neoplasia as a cause of death in 5 of 105 (5%) dogs.^32^ Examples included a splenic hemangiosarcoma with secondary hemoabdomen and a chemodectoma causing space-occupying effect and heart failure.^32^ The hemoabdomen caused by the hemangiosarcoma would have resulted in hypovolemic shock, making the cardiovascular system the immediate cause of death in this animal. Currently, no clear guidelines exist for classifying cause of death in PAD cases. Future autopsy PAD studies would benefit from clear criteria on classifying the different causes of death.

Unfortunately, human error (anesthetic or surgical) also can lead to PAD. In human medicine, up to 75% of PADs were reported as preventable and secondary to human error, such as medication overdose, equipment problems, or failed intubation.^41,69,119^ Often, a factor associated with these errors is a failure of communication. For example, the anesthetist is not informed of relevant clinical history by the surgical team.^41^ Given the variations in the standard of care in veterinary anesthesia, it seems likely that human error may play a role. However, without a standard reporting method, it is difficult to accurately assess this role. One study used voluntary critical incident reporting in 130 European veterinary practices.^106^ Of 2,155 incident reports, 40% of incidents led to patient harm. Of these, 55% were related to anesthesia and included death.^106^ Reported human errors in veterinary studies include equipment failure, failure to intubate, and medication overdose.^29,88,106^ Several case reports identified accidental injection of air into the vascular system (i.e., venous air embolism) as the cause of death.^49,84,113,118^ There are 2 questions that make an air embolism diagnosis challenging. First, was the event recognized when it occurred, and second, was the lesion associated with air embolism identified at autopsy? Potentially, diagnostic procedures, such as incising the right cardiac ventricle underwater are not commonly performed during veterinary autopsies; therefore, the accuracy of this test for confirming venous air embolism in veterinary species is unknown.^32^ Additionally, knowing the significance of identifying air bubbles remains a challenge; both the volume present relative to the animal’s size and the preservation of the body can influence interpretation and presence, respectively.

### The value of postmortem examination in perianesthetic deaths

Despite the overall decline in the use of autopsy in human PAD cases (from 50% in the 1950s to 2.4% in the early 2000s), studies continue to show the value of autopsies in helping reach clinical diagnosis for various diseases or complications, including PAD.^26,85,108^ In a study investigating the value of autopsy in the medicolegal defense of anesthesiologists, 50% of 980 cases reviewed had unexpected pathology diagnoses and 61% identified significant non-anesthetic contributions to death.^67^ The authors concluded that autopsy helped in the defense of anesthesiologists in 55% of claims.

Human studies offer a structured and standardized approach to the investigation of PADs.^94,112^ Of note is the recommendation of creating an anesthesia-surgical death committee. An independent anesthesiologist and surgeon review all medical records to reconstruct the course of events preceding the anesthetic death. Records include an autopsy report performed by a board-certified pathologist.^94^ The goal of combining these areas of expertise is to offer a greater understanding of pathophysiology leading to death and to help improve future patient care.

A consistent challenge in veterinary PAD autopsy studies has been a reliance on non-standardized clinical history collection methods and autopsy procedures.^32,43,104,105^ Given that medico-legal cases of PAD represented 211 of 1,706 (12.4%) legal case submissions to a Canadian diagnostic laboratory, creating a more standardized approach to the postmortem examination of these cases is important.^75^ The main goal of the autopsy is to rule out preexisting diseases or complications associated with the procedure or anesthesia that may have contributed to, or caused, death.^33^ Proposed procedures for perianesthetic death postmortem examination have been published, including a proposed checklist.^33,105^

An interesting trend identified in the multispecies autopsy study was that 174 of 221 (79%) submissions came from general practices, and 158 of 221 (71%) were undergoing elective procedures.^32^ General practices typically perform more elective procedures on healthier patients compared with referral or specialty practices; thus, one might expect a lower incidence of anesthetic complications. However, several factors may contribute to this submission bias, including the fact that general veterinary practices outnumber referral or teaching institutions. Additionally, owners may be more likely to submit their animal for autopsy if the animal was considered healthy before anesthesia, compared with an animal that had a significant underlying disease undergoing emergency surgery. Most cases lacking autopsy lesions to explain the cause of death were “young, healthy and undergoing elective procedures.”^32^ This raises the question: Why are lesions sometimes absent? One possibility is that pathologists may be missing subtle lesions. However, this seems unlikely, as these studies often involve multiple board-certified pathologists from various institutions. Another possibility is the acute nature of the incident, which may not allow sufficient time for lesions to develop, especially considering that detectable histologic signs of hypoxic cell death typically require at least 4 h to appear.^86^ Ideally, standardizing investigation of PADs, including consistent clinical history documentation, postmortem examinations, and collaboration with anesthesiologists and surgeons, would allow for a more thorough understanding of the condition and potentially help identify significant contributing factors.

## Challenges and limitations associated with perianesthetic death autopsies

### Lesions associated with cardiopulmonary resuscitation

In human medicine, the most reported CPR-related injuries are rib and sternum fractures, with elderly patients at highest risk and children rarely experiencing these complications.^31^ Lesions in abdominal organs are rarely reported, but are more likely to occur in patients with preexisting disease in these organs.^31^ Factors that increase the likelihood of CPR-related lesions in human medicine include the duration of CPR, the patient’s age, and the experience of the medical personnel.^31^

Lesions associated with veterinary CPR are frequent, with 1 autopsy study reporting that 34% of submitted domestic animal cases had lesions.^32^ In veterinary medicine, no clear consensus exists as to what constitutes a CPR lesion; exhaustive lists of lesions associated with CPR have been reported.^32,76,93,105^ These include multifocal hemorrhage in organs, such as the heart, lungs, diaphragm, intercostal muscles, liver, gall bladder, and thymus; effusions (both hemorrhagic and non-hemorrhagic) in body cavities, including the pericardium, thorax, and abdomen; various lung lesions, such as congestion, edema, and atelectasis; as well as pneumothorax, gastric bloat, and myocardial necrosis.^32,76,93,105^ Lung hemorrhage tends to be one of the most frequently reported lesions, and even mild hemorrhage should be considered CPR-related.^76,105^ Rib fractures in veterinary patients are rarely reported and are more likely to occur in older animals—a finding that pathologists should note, as it could indicate underlying bone conditions.^32,76,93^ One study investigated CPR in a university teaching hospital; none of the 62 animals that had CPR performed (and subsequently underwent autopsy) had pneumothorax. This highlights the importance of characterizing lesions reported in CPR veterinary patients at autopsy.^76^ A checklist of potential CPR-associated lesions should be considered part of a standardized autopsy report.

### Complications without lesions

Unfortunately, many PADs lack specific lesions, and a significant portion of PAD studies classify the cause of death as “unknown” in both clinical and autopsy studies.^19,32,43,104,105^ In dogs, an unknown cause of death is reported in 20–40% of the clinical studies, and in 1 autopsy study, an unknown cause of death was reported in 43% of cases.^20,32,38,96^ Three of the deaths lacking postmortem lesions in the autopsy study had a history of anesthetic complications, such as a closed adjustable pressure-limiting valve, which presumably could cause barotrauma to the lungs. In cats, 8–65% of clinical PAD studies and 26–63% of autopsy PAD studies report an unknown cause of death.^19,32,38,43,95,105,107,120^

### Lack of clinical history

Clinical history offers essential context for pathologists, particularly in PAD cases. However, clinical histories on submission forms were incomplete in 19–88% of pathology studies. In addition, no standardized criteria exists for what constitutes an adequate clinical history.^16,32,104^ In the multispecies autopsy study, the authors reported that key information was often missing from the submission form, including the animal’s clinical status at the time of surgery (e.g., ASA status was recorded in only 1 of 221 submissions), the type of procedure performed, the time of death, and whether CPR was administered.^32^ The neuter autopsy study was the first to recommend specific information for inclusion in PAD submission forms (Table 1).^43^ Clinical history can potentially introduce biases, particularly if incomplete. To mitigate these biases, pathologists could implement a standardized submission form to guide clinicians in providing essential information (Table 1).

For PAD cases, being aware of the procedure performed, and its potential complications will help the pathologist identify which organ systems should be the focus of their examination. Pathologists should also be aware of the specific complications associated with commonly performed procedures. For example, neuter surgeries may result in hemorrhage, aspiration, surgical site dehiscence, or gossypiboma, whereas dental procedures can lead to fractures, retained root tips, or salivary duct injury.^3,5,7,14,64,90^ Although not all complications are fatal, identifying such lesions helps rule out any potential surgical complications that may have contributed to death.

The animal’s pre-procedure health status is an important part of submission history. If the animal was deemed high-risk and assigned a high ASA status, the clinician should provide reasoning. Details on preexisting disease(s), ongoing treatments, and results from additional diagnostic tests (e.g., bloodwork) are important to include. For example, knowledge that a dog with idiopathic epilepsy is being treated with phenobarbital can alert the pathologist to examine for potential liver disease. Signalment is also important, as noted with brachycephalic breeds.^48,70,71^ Ancillary testing results and clinical assessments made by the practitioner can help identify the stability of the animal before the procedure. Vaccine and deworming history are important to understand the potential risk for infectious diseases. The autopsy study from the neuter clinics in New York state identified that <10% of the feline PAD cases were infected with the lungworm *Aelurostrongylus abstrusus*, which resulted in lung disease.^43^ Confirming if the animal was fasted, and for how long, might indicate whether aspiration of gastric contents was a risk factor.

Description of the surgical procedure, and any complications encountered or associated with surgery, will direct the pathologist to these regions. For example, examination of the integrity of sutures within the ovarian pedicles and uterine stump is essential in ovariohysterectomy cases.^32,105^ Details on complications encountered that were not immediately fatal (e.g., aspiration pneumonia) or non-fatal during anesthesia should also be reported. For example, were there any episodes of regurgitation, difficulties with tracheal intubation, hypothermia, or hypotension? If multiple attempts to intubate were required, the pathologist would closely inspect the laryngeal and tracheal mucosa for evidence of trauma. In human medicine, medical devices are recommended to be kept in place (e.g., endotracheal tube, urinary catheter) to allow the pathologist to fully examine these regions, a practice not currently standard in veterinary medicine.^62,112^ Prolonged hypotension would direct investigation of organs for indications of ischemia, particularly the kidneys and brain. Hypothermia—a common complication in veterinary anesthesia—can be fatal if severe.^103^

A description of the stage of anesthesia when death occurred and if specific procedures were performed around the time of the complication are important to document. For example, an injection given a few minutes before cardiac arrest could represent a medication error or anaphylaxis.^32^ If death occurred during recovery when the trachea was not intubated, the oropharynx and laryngeal region should be examined for signs of obstruction.

A description of CPR, if performed, will help correlate certain lesions with the act of CPR. For example, if intracardiac epinephrine was injected, cardiac lesions (such as pericardial hemorrhage) might be expected. In summary, the more details collected in the clinical history, the more accurate the pathologist can be in interpreting identified lesions.

### Procedures not performed routinely

Several procedures not routinely performed in veterinary medicine could provide valuable information regarding the cause of PAD. Although interpretation of these additional data may require input from other specialists, such information could be especially useful in cases in which no significant autopsy lesions are identified.

Autopsy serum chemistry and toxicology testing are not performed routinely in veterinary medicine.^33^ Although many tests are widely available in human medicine, few are validated in veterinary species.^102^ However, toxicologic investigation is particularly relevant in PAD cases, given that many toxicants do not produce specific autopsy lesions, and human error (such as accidental overdose) is a common cause of PAD in human medicine.^41,62,69,119^ Identifying toxicologic causes of death overall can be challenging. However, in veterinary medicine, the range of administered drugs in PAD cases is typically narrow and should be evident from a detailed clinical history. In human forensic pathology, standardized toxicologic sampling is recommended in all PAD investigations and generally includes femoral blood, gastric contents, urine, vitreous humor, and ~10 g each of brain, lung, liver, kidney, and subcutaneous fat.^62^ Incorporating toxicologic testing into veterinary PAD postmortem examination should be considered to improve diagnostic yield, particularly in cases that lack distinct lesions. Currently, toxicologic testing in veterinary medicine is limited, and interpretation of results is challenging given the frequent lack of species-specific reference intervals.

Diagnostic imaging and electrocardiography (ECG) also offer information that can lead to a better understanding of PAD cases. Full-body radiographs or computed tomography, particularly of the thorax, are recommended before postmortem examination to help identify lesions, such as fractures, pneumothorax or venous air embolism.^32,33^ However, radiographs may not be feasible because of logistical (e.g., the animal is frozen or in rigor mortis) or biosecurity constraints. Performing diagnostic imaging at the clinic has 2 advantages—easier access to equipment (not always available in a pathology laboratory) and timing, as images can be obtained before autopsy changes (such as freezing or rigor mortis) take effect. ECG may also provide information from the time of death without leaving visible lesions. Future submission forms might require that diagnostic imaging performed prior to death, as well as any available ECG recordings obtained while the animal was alive, be included with the submission. This would allow radiologists or cardiologists to review the images, supporting a multidisciplinary approach to these investigations.

### Limitations of PAD investigations

Current procedures used to investigate cases of PAD in veterinary medicine have several limitations. In human medicine, cases of PAD that undergo legal trial often have an autopsy report from a pathologist in addition to a review of all clinical history by an independent anesthesiologist and/or surgeon.^67^ Human studies emphasize the importance of understanding the pathophysiology of events preceding death by having experts closely examine all documentation.^94,112^ In veterinary medicine, such collaborative investigations are rare. Only 2 studies report using an independent panel of surgeons and anesthetists to review cases and determine the cause of death; however, autopsies were rarely part of this review process.^19,57^ Having an anesthesiologist review the histories of PAD autopsy submissions may help in determining the cause of death, as was previously suggested.^32^ Although this type of multidisciplinary review may increase the time and cost of a complete PAD investigation, it offers a best-practice approach that is particularly valuable in cases with potential medicolegal implications or when no clear link can be identified between the autopsy findings and cause of death.

We have summarized what would be the gold standard approach to investigating cases of PAD in veterinary medicine. However, we also recognize that limitations and differences exist in veterinary compared with human medicine. First, in human medicine, cost is often subsidized, and many procedures are in place to help reduce risk. In veterinary medicine, additional expenses could include consultation with an anesthesiologist. Despite this limitation, it is useful to make recommendations on how these cases should be worked up and to acknowledge that relying solely on autopsy for a diagnosis is insufficient in many cases. Although all these recommendations may not be feasible in every case, striving toward improved PAD investigation holds promise for increasing the proportion of cases in which a diagnosis is achieved. Preventable deaths might be further reduced by what we learn from PAD cases.

## Supplemental Material

sj-pdf-1-vdi-10.1177_10406387261421223 – Supplemental material for Perianesthetic death in dogs and cats: a scoping review Supplemental material, sj-pdf-1-vdi-10.1177_10406387261421223 for Perianesthetic death in dogs and cats: a scoping review by Nicole Rose, Bruce Wobeser and Daniel J. Pang in Journal of Veterinary Diagnostic Investigation
