# Supplementary material for: Perianesthetic death in dogs and cats: a scoping review
Source: J Vet Diagn Invest. 2026 Feb 17:10406387261421223. Online ahead of print. doi: 10.1177/10406387261421223 (PMC12916337; doi:10.1177/10406387261421223)
Supplement: sj-pdf-1-vdi-10.1177_10406387261421223 – Supplemental material for Perianesthetic death in dogs and cats: a scoping review [file sj-pdf-1-vdi-10.1177_10406387261421223.pdf]

JVDI: Supplemental material

Rose N, et al. Perianesthetic death in dogs and cats: a scoping review

Supplemental Table 1. Comparison of the current American Society of Anesthesiologist physical status (ASA) classification definition and examples in veterinary and human medicine.[10,23]

ASA	Physical status with examples in veterinary medicine	Physical status with examples in human medicine
I	Normal healthy patients (e.g., no discernible disease; elective surgeries including ovariohysterectomy or castration).	A normal healthy patient (e.g., healthy, non-smoking, no or minimal alcohol use).
II	Patient with mild systemic disease (e.g., skin tumor, fracture without shock, uncomplicated hernia, cryptorchidectomy, localized infection, or compensated cardiac disease).	A patient with mild systemic disease (e.g., mild diseases only without substantive functional limitations).<br>Current smoker, social alcohol drinker, pregnancy, obesity (30<BMI<40), well-controlled DM/HTN, mild lung disease.
III	Patients with severe systemic disease (e.g., fever, dehydration, anemia, cachexia, or moderate hypovolemia).	A patient with severe systemic disease (e.g., substantive functional limitations; one or more moderate-to-severe diseases; poorly controlled DM or HTN, COPD, morbid obesity [BMI ≥40], active hepatitis, alcohol dependence or abuse, implanted pacemaker, moderate reduction of ejection fraction, ESRD undergoing regularly scheduled dialysis, history [>3 mo] of MI, CVA, TIA, or CAD/stents).
IV	Patients with severe systemic disease that is a constant threat to life (e.g., uremia, toxemia, severe dehydration and hypovolemia, anemia, cardiac decompensation, emaciation, or high fever).	A patient with severe systemic disease that is a constant threat to life (e.g., recent [<3 mo] MI, CVA, TIA or CAD/stents, ongoing cardiac ischemia or severe valve dysfunction, severe reduction of ejection fraction, shock, sepsis, DIC, ARD or ESRD not undergoing regularly scheduled dialysis
V	Moribund patients not expected to survive 24 h with or without operation (e.g., extreme shock and dehydration, terminal malignancy or infection, or severe trauma).	A moribund patient who is not expected to survive without the operation (e.g., ruptured abdominal/thoracic aneurysm, massive trauma, intracranial bleed with mass effect, ischemic bowel in the face of significant cardiac pathology or multiple organ/system dysfunction).
VI	Not applicable.	A declared brain-dead patient whose organs are being removed for donor purposes.
E	Denotes emergency and can be added to any of the above classes that require immediate intervention or surgery.	Denotes emergency surgery = delay in treatment of the patient would lead to a significant increase in the threat to life or body part.

ARD = airway respiratory disease; BMI = body mass index; CAD = coronary artery disease; COPD = chronic obstructive pulmonary disease; CVA = cerebrovascular accident; DIC = disseminated intravascular coagulation; DM = diabetes mellitus; ESRD = end-stage renal disease; HTN = hypertension; MI = myocardial infarction; PCA = patient-controlled analgesia; TIA = transient ischemic attack.

Supplemental Table 2. Studies investigating perianesthetic death rates in dogs.

Albrecht and Blakely[6]: Anesthetic mortality: A 5-y survey of the records of the Angell Memorial Animal Hospital

Design: Survey of records over last 5 y. Single center: referral United States. 1946−1950.

Definition: All hospital cases (excluding out-patient cases) in which death occurred any time from induction until the patient either returned to consciousness or to their preoperative condition. This includes deaths attributable to the anesthesia, the surgical procedure, the condition of the patient, or a combination of these factors.

Results: 21,597 animal anesthesias. 14,640 dog anesthesias. Death risk in dogs: 0.259% (*n* = 38). Total death risk: 0.295% (*n* = 64).

RF: Compared different anesthetic agents, but no conclusions made.

AU: 2.6% reported (1/38). COD: Summarized for all species, 92.2% reported (59/64).

Clarke and Hall[29]: A survey of anaesthesia in small animal practice: AVA/BSAVA report

Design: Prospective survey. Multicenter: 53 GP. United Kingdom. 1984–1986.

Definition: Not reported.

Results: 20,814 anesthesias in dogs and cats. Death risk: 0.23% (*n* = 48). Death risk healthy: 0.11% (*n* = 23). Death risk sick: 3.12% (*n* = 25).

RF: Xylazine, ASA classification, animals not closely monitored.

AU: The authors report "if available autopsy findings were recorded"; however, it is not clear which cases had AUs performed. COD: 85% reported (~41/48).

Dodman and Lamb[36]: Survey of small animal anesthetic practice in Vermont

Design: Retrospective survey. Multicenter: 39 GP. United States. Dec 1989.

Definition: "How many animals do you believe have died as a result of anesthesia in the last two years?"

Results: 30,000 dog anesthetics/y (estimated). RR: 46.5% (41/88). Death risk: *0.055% (*n* = 33). *The authors report death risk: 0.11% (*n* = 33); however, the 33 deaths were over 2 y, and the authors estimated ~30,000 anesthesias/y. Therefore, 33 deaths per 60,000 anesthesias would equal death risk: 0.055% (*n* = 33).

RF: Not reported. AU: Not reported. COD: Not reported.

Howe[56]: Short-term results and complications of prepubertal gonadectomy in cats and dogs

Design: Design not reported. Multicenter: 2 spay-neuter. United States. 18-mo period, do not state the dates.

Definition: All complications (including death) in cats and dogs undergoing spay-neuter in association with the 4th-year surgical teaching program with the veterinary school. Compared groups based on age: group 1, <12 wk; group 2, 12−23 wk; group 3, ≥24 wk. Follow-up period not specified, but some deaths occurred up to 7-d post-procedure.

Results: 1,988 animal anesthesias (1,213 dog anesthesia; 775 cat anesthesia). Death risk: 0.8% (*n* = 16). Deaths were reported for cats and dogs together.

RF: Not reported. AU: 12.5% reported (2/16). COD: 100% reported (16/16).

Dyson et al.[38]: Morbidity and mortality associated with anesthetic management in small animal veterinary practice in Ontario

Design: Prospective cohort. Multicenter: 66 GP. Canada. Jan−Jul 1993.

Definition: Perioperative deaths from cardiac arrest, follow-up period not specified.

Results: 8,087 anesthesias in dogs. Death risk: 0.11% (*n* = 9). Death risk in healthy: 0.067% (*n* = 5).

RF: Xylazine, ASA classification ≥III. AU: 11% reported (1/9). COD: 100% reported (9/9).

Hosgood and Scholl[55]: Evaluation of age as a risk factor for perianesthetic morbidity and mortality in the dog

Design: Prospective cohort. Single center: teaching. United States. Jun 1995−Jul 1996.

Definition: Dogs ≥6-mo-old that underwent inhalation anesthesia of at least 30 min duration with death or euthanasia up to 24 h after the end of anesthesia. All deaths, independent of cause, were included.

Results: 942 anesthesias in dogs. Death risk: 2.45% (*n* = 23) *includes intraoperative euthanasia. Anesthetic deaths: 1.49% (*n* = 14).

RF: Old age (based on breed), high ASA classification. AU: Not reported. COD: 79 serious complications reported, unclear which resulted in death.

Gaynor et al.[42]: Complications and mortality associated with anesthesia in dogs and cats

Design: Prospective. Single center: teaching. United States. Feb 1993−Jan 1994.

Definition: "Death intra-anesthesia, during recovery, or within 24 h of anesthesia."

Results: 2,556 anesthesias in dogs. Deaths risk: 0.43% (number not reported, but Brodbelt[17] reported number to be 11 dogs).

RF: Not reported. AU: Not reported. COD: Unclear.

Joubert[60]: Routine veterinary anaesthetic management practices in South Africa

Design: Retrospective questionnaire. Multicenter: referral and GP. South Africa. Feb−Apr 1999.

Definition: "Patients dying under anesthesia per year."

Results: 235,456 anesthesias in dogs and cats (estimated). RR: 27% (162/600). Death risk in dogs and cats: 0.08% (*n* = 190; estimated).

RF: Not reported. AU: Not reported. COD: Not reported.

Joubert[61]: Anaesthesia and analgesia for dogs and cats in South Africa undergoing sterilization and with osteoarthritis—an update from 2000

Design: Retrospective, questionnaire. Multicenter: referral and GP. Jul−Dec 2005.

Definition: "the number of animals that died under anesthesia per year."

Results: 500,000 anesthesias in dogs (estimated). RR: 18.3% (109/600). Death risk in dogs and cats: 0.099% (estimated).

RF: Not reported. AU: Not reported. COD: Not reported.

Brodbelt et al.[17]: Risk factors for anaesthetic-related death in referred dogs

Design: Retrospective nested case-control. Single center: teaching. United Kingdom. Feb 1999−Apr 2002.

Definition: "Anesthetic-related deaths occurring within 48 h of anaesthesia in which anaesthesia could not be excluded from contributing to death."

Results: 6,026 anesthesias in dogs. Death risk Apr 2001–2002: 0.58% (*n* = 20).

RF: high risk status. Reduced risk: acepromazine in pre-medication. AU: Not reported. COD: Not reported.

Redondo et al.[95]: Normal values and incidence of cardiorespiratory complications in dogs during general anaesthesia. A review of 1,281 cases

Design: Retrospective. 2 centers: teaching. Spain. Sep 1998−Jul 2004.

Definitions: Deaths during the anesthesia and 48 h after extubation. Exclusion: dogs that were killed.

Results: 1,281 anesthesias in dogs. Death risk: 0.94% (*n* = 12).

RF: Not reported. AU: Not reported. COD: 100% reported (12/12).

Brodbelt et al.[19]: The risk of death: the confidential inquiry into perioperative small animal fatalities

Design: Prospective cohort with nested case-control. Multicenter: 117 GP and referral. United Kingdom. Jun 2002−2004.

Definition: "perioperative death (including killing) occurring after pre-medication and within 48 hours of termination of the procedure, except where death or killing was due solely to inoperable surgical or pre-existing medical conditions."

Results: 98,036 dog anesthetics/sedations. Death risk: 0.17% (n = 163). Death risk in healthy: 0.05% (49/90,618). Death risk in sick: 1.33% (99/7418).

RF: Dogs lower risk than cats and rabbits. Post-operative period most common time for death.

AU: 10% reported, no details provided. COD: 91% reported (148/163). classified by an independent review panel of veterinary anesthetists and surgeons using a specified list of criteria.

Brodbelt et al.[20]: Results of the Confidential Enquiry into Perioperative Small Animal Fatalities regarding risk factors for anesthetic-related death in dogs

Design: Case-control. Multicenter: 117 GP and referral. United Kingdom. Jun 2002−2004.

Definition: "death or euthanasia within 48 h after termination of anesthesia or sedation, except when death was due solely to the surgical procedure or pre-existing condition."

Results: 98,036 dog anesthetics/sedations. Death risk: 0.15% (n = 148).

RF: Increased ASA classification, urgency of procedure, age, decreased body weight, major vs. minor procedure, use of injectable agents for anesthetic induction and halothane for maintenance or inhalant anesthetics alone. AU: Reported in Brodbelt et al.[19] COD: Reported in Brodbelt et al.[19]

Bille et al.[12]: Risk of anaesthetic mortality in dogs and cats: an observational cohort study of 3,546 cases

Design: Prospective cohort. Single center: referral. France. Apr 2008−2010.

Definition: Alive or dead at end of anesthesia defined as return of consciousness, rectal temperature >36°C, ability to maintain sternal recumbency. Exclusion: animals sedated or euthanized.

Results: 3,546 dog and cat anesthesias. Death risk: 1.35% (n = 48). Death risk in healthy: 0.12% (3/2,602). Death risk in sick: 4.77% (45/944).

RF: Poor health status. No risk associated with species or age. AU: Not reported. COD: Not reported.

Gil and Redondo[44]: Canine anaesthetic death in Spain: a multicentre prospective cohort study of 2012 cases

Design: Retrospective. Multicenter: 2 teaching. Spain. Sep 1998−Jul 2004.

Definition: "Deaths which occurred during the anesthesia and 48 h after extubation."

Results: 2,012 dog anesthesias, another 12 euthanized due to severity of lesions. Death risk: 1.29% (n = 26). Death risk in healthy: 0.33% (4/1,495). Death risk in sick: 4.06% (21/517).

RF: High ASA classification. Reduced risk with use of analgesics. AU: Not reported. COD: Not reported.

Bille et al.[13]: An evidence-based medicine approach to small animal anaesthetic mortality in a referral practice: the influence of initiating three recommendations on subsequent anaesthetic deaths

Design: Prospective cohort. Single center: referral. France. Jun 2010−Aug 2011.

Definition: Alive or dead at end of anesthesia defined as return of consciousness, rectal temperature >36°C, ability to maintain sternal recumbency. Exclusion: animals sedated or euthanized.

Results. 2,685 dog and cat anesthesias. Death risk: 0.8% (21/2685). Death risk in healthy: Not reported. Death risk in sick: 2.2% (18/834)—an improvement from period 1 (Bille, 2012) where deaths overall: 1.35% (48/3546), deaths in sick: 4.8% (45/944).

RF: old age, poor health status. AU: Not reported. COD: Not reported.

McMillan and Darcy[78]: Adverse event surveillance in small animal anaesthesia: an intervention-based, voluntary reporting audit

Design: Prospective, voluntary reporting. Single center: teaching. United Kingdom. Jun 2012−Jun 2013.

Definition: Voluntary reporting of adverse events over 1 y. No clear definition, death during the anesthesia. The definition/cause of euthanasia is not described.

Results: Total anesthesia: 1,386. Total death risk: 0.43% (n = 6). Euthanasia: 5 cases. The study does not distinguish between amount of deaths/euthanasia in dogs and cats. 972 dog anesthesias. Overall compliance: 80.4%.

RF: Cats were more likely to have adverse events than dogs. AU: Not reported. COD: Not reported.

Itami[57]: Association between preoperative characteristics and risk of anaesthesia-related death in dogs in small-animal referral hospitals in Japan

Design: Prospective cohort. Multicenter: referral and teaching. Japan. Apr 2010−Mar 2011.

Definition: Death occurring within 48 hours after extubation. Exclusion: death from surgical error or euthanasia.

Results: 4,310 anesthesias in dogs (7 surgical errors and 6 euthanasia were excluded). Death risk: 0.65% (n = 28). 75% had preexisting disease.

RF: low serum glucose, disturbance of consciousness, elevated WBCs, ASA classification ≥III. AU: Not reported. COD: 100% reported (28/28). Classified by independent review panel.

Levy et al.[68]: Perioperative mortality in cats and dogs undergoing spay or castration at a high-volume clinic

Design: Retrospective. Single center: spay-neuter. United States. 2010−2016.

Definition: "Death occurring in the 24 h period starting with the administration of the first sedation or anesthetic drugs." Cats and dogs undergoing spay-neuter surgery.

Results: 42,349 anesthesias in dogs. Death risk: 0.009% (n = 4).

RF: Higher risk in females than males. higher risk in cats than dogs. AU: Not reported. COD: Not reported.

Matthews et al.[74]: Factors associated with anesthetic-related death in dogs and cats in primary care veterinary hospitals

Design: Retrospective matched case-control. Multicenter: 822 GPs. United States. 2010−2013.

Definition: Banfield hospitals where death or euthanasia within 7 d of an anesthetic episode for which anesthesia or sedation could not be reasonably ruled out as contributing to death.

Results: 1,817,193 dog anesthesias. Death risk: 0.05% (n = 982).

RF: underweight, increasing age, non-elective procedures, pre-anesthetic physical exam not performed, hematocrit outside reference. AU: Not reported. COD: Not reported.

Smith et al.[111]: Retrospective study of intra-anesthetic predictors of prolonged hospitalization, increased cost of care and mortality for canine patients at a veterinary teaching hospital

Design: Retrospective observational. Single center: teaching. United States. 2007−2014.

Definition: "General anesthesia is defined here as a drug-induced unconsciousness characterized by a controlled and reversible depression of the central nervous system and analgesia sufficient to allow endotracheal intubation."

Results: 235 dog anesthesias. Death risk: 5.1% (n = 12).

RF: ASA classification, administration of colloids. AU: Not reported. COD: Not reported.

Kreisler et al.[64]: Outcomes of elective gonadectomy procedures performed on dogs and cats by veterinary students and shelter veterinarians in a shelter environment

Design: Retrospective, cohort. Single center: spay-neuter. United States. June 2012−Sept 2014.

Definition: Compared the complication rates between veterinary students (VS) and shelter employed veterinarian (SEV) performing spay/neuter procedures on dogs and cats from anesthetic induction to 72 h after the procedure.

Results: 2,374 dog anesthesias (VS: 897 dog anesthesias; SEV: 1,477 dog anesthesias). Death risk in dogs: 0.084% (n = 2). Death risk VS: 0% (n = 0/897). Death risk SEVL: 0.135% (n = 2/1,477). Total anesthesia (dog and cat): 10,073 total anesthesias. Total death risk: 0.238% (n = 24). Death risk VS: 0.13% (n = 4/3,048). Death risk SEV: 0.285% (n = 20/7,025).

RF: Not reported. AU: Not reported. COD: 100% reported (2/2).

Shoop-Worrall et al.[110]: Mortality related to general anaesthesia and sedation in dogs under UK primary veterinary care
Design: Prospective nested case-control. Multicenter: >300 GP and referral. United Kingdom. Jan 2010−Dec 2013.
Definition: Dogs undergoing general anesthesia/sedation recorded where death due to the anesthetic could not be excluded, within 48 h and 2 wk of the event.
Results: 57,318 dog anesthesias/sedations. Death risk within 48 h: 0.10% (*n* = 159). Death risk within 2 w: 0.14% (*n* = 219). Death risk in spay-neuters: 0.009% (*n* = 8).
RF: Greater age, poor ASA classification, more urgent procedures. AU: Not reported. COD: 100% reported (219/219).
Morrison et al.[82]: Development and implementation of veterinary anesthesia medical quality standards for primary care
Design: Qualitative observation with clinical auditing. Multicenter: unclear on total number but there are ~1,000 Banfield hospitals, GP. United States. Nov 2017−Jan 2020.
Definition: "…any death that occurred within 7 d of a documented general anesthetic procedure." Risk was reassessed after implementing medical quality standards through clinical auditing.
Results: Nov 2017: 69,315 anesthesias. Death risk: 0.074% (*n* = 51). Jan 2020: 80,162 anesthesias. Death risk: 0.045% (*n* = 36). Overall: 2,038,318 anesthesias. Overall death risk: 0.056% (*n* = 1,136).
RF: Not reported. AU: Not reported. COD: Not reported.
Lutvikadic and Maksimovic[73]: Use of ketamine and xylazine anesthesia in dogs: a retrospective cohort study of 3,413 cases
Design: Retrospective cohort using intermittent injectable ketamine and xylazine intravenous anesthesia. Single center: teaching. Bosnia and Herzegovina. 2019.
Definition: "dogs undergoing general anesthesia for elective ovariohysterectomy and orchiectomy" and "all dogs underwent observation for 48 h postoperatively." Only deaths that could not be explained by preexisting medical conditions or intraoperative complications. All dogs were ASA classification I or II. Brachycephalic breeds were excluded.
Results: 3,413 dog anesthesias. Death risk within 48 h: 0.15% (*n* = 5).
RF: Level of experience of the anesthesiologist. AU: Not reported. COD: Not reported.
Oda et al.[87]: Perianesthetic mortality in English Bulldogs: a retrospective analysis in 2010–2017
Design: Retrospective cohort comparing English Bulldogs (EB) to other brachycephalics (OB) and non-brachycephalics (NB). Single center: teaching. United States. Jan 2010−Sept 2017.
Definition: 1) Total deaths: whether the EB patient was discharged from the hospital (survivor) or died during hospitalization (non-survivor), regardless of cause of death. 2) Anesthesia-related death: whether EB patient died from a cause related to anesthesia, defined as a death within 48 h after anesthesia unless the death was unrelated to anesthesia.
Results: 229 EB anesthesias (218 OB anesthesias; 229 NB anesthesias). Total death risk for EB: 6.6% (*n* = 15). Anesthesia-related death risk for EB: 3.9% (*n* = 9).
RF: interventional procedure, acepromazine during recovery, gastrointestinal comorbidities.
AU: Not reported. COD: 100% reported (15/15).
Varkoulis et al.[117]: A retrospective study on canine and feline mortality during anaesthesia at a university clinic in Greece
Design: Retrospective cohort. Single center: teaching. Greece. Jan 2018−Dec 2019.
Definition: "any death that occurred during anaesthesia, i.e. between induction of general anesthesia with an injectable or volatile anaesthetic agent and cessation of administration of these agents." Animals receiving sedation were also included. Exclusion criteria: insufficient data, euthanasia or if the animal had >1 anesthesia.

Results: 1,187 dog anesthesias. Death risk 0.6% (*n* = 7; cardiac arrest 1.1%). ASA classification I or II 0.1%; ASA classification III–V 2.6%.
RF: ASA classification, anesthetic protocol, use of inotropes. AU: Not reported. COD: Not reported.
Redondo et al.[96]: Anesthetic mortality in dogs: a worldwide analysis and risk assessment
Design: Observational prospective cohort. Multicenter: 405 GP, referral and teaching. Spain, Argentina, France, United Kingdom, United States, Chile, Portugal, and Australia. Feb 2016−Dec 2022.
Definition: Dogs undergoing anesthesia where death was between preanesthetic medication and 48 h extubation. Deaths were solely associated with anesthesia, excluding those related to euthanasia due to preexisting lesions and deaths due to medical/surgery reasons.
Results: 55,022 dog anesthesias. Death risk: 0.69% (*n* = 378).
RF: greater age, poor ASA classification, obesity. AU: If a dog died, more details were obtained including any postmortem examination results; however, results were not reported. COD: Not reported.

ASA = American Society of Anesthesiologists physical status classification; AU = autopsies, if reported, the percentage of deaths that had an autopsy performed; COD = cause of death = the percentage of all deaths in which a cause of death was reported; Death in healthy = percentage of deaths in ASA I or II animals; Death in sick = percentage of deaths in ASA III–V animals; Death risk = total number of deaths out of all anesthetic procedures; Design = study design and the number and types of centers included; Definition = definition of perianesthetic death, including length of follow-up and any inclusion or exclusion criteria, if reported; DIC = disseminated intravascular coagulation; GP = general practice; Referral = specialty practices; Results = total number of anesthetic procedures. Questionnaire response rates (percentage of number returned out of total). RF = major risk factors associated with perianesthetic death; RR = response rate; Spay-neuter = facilities performing only de-sexing procedures; teaching = university teaching hospital.

Supplemental Table 3. Studies investigating perianesthetic death rates in cats.

Albrecht and Blakely[6]: Anesthetic mortality: A five-year survey of the records of the Angell Memorial Animal Hospital

Design: Survey of records over last 5 y. Single center: referral United States. 1946−1950.

Definition: All hospital cases (excluding out-patient cases) where death occurred any time from induction until the patient either returned to consciousness or to their preoperative condition. This includes deaths due to either the anesthesia, the surgical procedure, the condition of the patient or a combination of these factors.

Results: 6,936 cat anesthesias. Death risk: 0.360 (*n* = 25). 21,597 animal anesthesias. Death risk: 0.295% (*n* = 64).

RF: Compared different anesthetic agents but no conclusions made. AU: Not report. COD: All species, 92.2% reported (59/64).

Dodman[35]: Feline anaesthesia survey

Design: Retrospective questionnaire. Multicenter: 29 GP. United Kingdom. 1975.

Definition: "How many cats in your practice died as a result of anaesthesia last year?"

Results: 11,227 cat anesthesias (estimate). RR: 24% (29/120). Death risk: 0.3% (*n* = 35). >75% considered young and healthy.

RF: Not reported. AU: Not reported. COD: Not reported.

Clarke and Hall[29]: A survey of anaesthesia in small animal practice: AVA/BSAVA report

Design: Prospective survey. Multicenter: 53 GPs. United Kingdom. 1984−1986.

Definition: Not reported.

Results: 20,103 cats anesthesias (estimated). Death risk: 0.29% (*n* = 59). Death risk in healthy: 0.18% (35/19,339). Death risk in sick: 3.3% (25/764).

RF: ASA classification, xylazine, endotracheal intubation. AU: The authors report "if available autopsy findings were recorded"; however, it is not clear which cases had AUs performed. COD: ~85% reported (50/59).

Dodman and Lamb[36]: Survey of small animal anesthetic practice in Vermont

Design: Retrospective survey. Multicenter: 39 GP. United States. Dec 1989.

Definition: "How many animals do you believe have died as a result of anesthesia in the last two years?"

Results: 32,000 cat anesthetics/y (estimated). RR: 46.5% (41/88). Death risk: *0.03% (*n* = 19). *The authors report death risk: 0.06% (*n* = 19); however, the 19 deaths were over 2 y, and the authors estimated ~32,000 anesthesias/y. Therefore, 19 deaths per 64,000 anesthesias would equal death risk: 0.03% (*n* = 19).[20]

RF: Cats lower death rate than dogs. AU: Not reported. COD: Not reported.

Howe[56]: Short-term results and complications of prepubertal gonadectomy in cats and dogs

Design: Design not reported. Multicenter: 2 spay-neuter. United States. 18-mo period, do not state the dates. Definition: All complications (including death) in cats and dogs undergoing spay-neuter in association with the 4th-year surgical teaching program with the veterinary school. Compared groups based on age: group 1, <12 wk; group 2, 12−23 wk; group 3, ≥24 wk. Follow-up period not specified, but some deaths occurred up to 7 d post-procedure.

Results: 1,988 animal anesthesias (1,213 dogs; 775 cats). Death risk: 0.8% (*n* = 16). Deaths were reported for cats and dogs together.

RF: Not reported. AU: 12.5% reported (2/16). COD: 100% reported (16/16).

Dyson et al.[38]: Morbidity and mortality associated with anesthetic management in small animal veterinary practice in Ontario

Design: Prospective, cohort. Multicenter: 66 GP. Canada. Jan−Jul 1993.

Definition: Perioperative deaths from cardiac arrest.

Results: 8,702 cat anesthesias. Death risk: 0.10% (*n* = 8). Death risk in healthy: 0.05% (*n* = 4). Complications: 1.3%.

RF: ASA classification, reduced risk if technician present. AU: 37.5% reported (3/8). COD: 100% reported (8/8).

Gaynor et al.[42]: Complications and mortality associated with anesthesia in dogs and cats

Design: Prospective. Single center: teaching. United States. Feb 1993−Jan 1994.

Definition: "Death intra-anesthesia, during recovery, or within 24 h of anesthesia."

Results: 683 cat anesthesias. Death risk: 0.43% (numbers not reported).

RF: Not reported. AU: Not reported. COD: Not reported.

Hosgood and Scholl[54]: Evaluation of age and American Society of Anesthesiologists physical status as risk factors for perianesthetic morbidity and mortality in the cat

Design: Prospective cohort. Single center: teaching. United States. Jun 1995−Jul 1996.

Definition: Cats ≥6-mo-old that underwent inhalation anesthesia of at least 30 min duration up to 24 h after the end of anesthesia. Euthanasia or death (demise of the animal due to any cause except euthanasia).

Results: 138 cat anesthesias. Death risk (including euthanasia): 5.1% (*n* = 7). Serious complications: 10.9% (*n* = 15).

RF: ASA classification, not age. AU: Not reported. COD: Not reported.

Scott et al.[107]: Characteristics of free-roaming cats evaluated in a trap-neuter-return program

Design: Cross-sectional. Single center: spay-neuter. United States. Jul 1998−Dec 2001.

Definition: "Necropsies were performed on all cats that were euthanized or died."

Results: 5,323 cat anesthesias. Unexpected death: 0.3% (*n* = 14). Euthanized: 0.4% (*n* = 24) due to poor prognosis.

RF: Not reported. AU: 100% reported with 64% having underlying disease. COD: 100% reported.

Williams et al.[120]: Use of the anesthetic combination of tiletamine, zolazepam, ketamine, and xylazine for neutering feral cats

Design: Retrospective and prospective. Multicenter: 2 spay/neuter. United States. Jul 1996−Aug 2000

Definition: Cats that died between the administration of the anesthetic and the time of discharge.

Results: 7,502 cat anesthesias. Death risk: 0.35% (*n* = 26). Deaths solely from anesthesia: 0.24% (*n* = 17).

RF: Not reported. AU: 100% reported with 35% having unexpected pathology. COD: 100% reported (26/26).

Brodbelt et al.[18]: Risk factors for anaesthetic-related death in cats: results from the confidential enquiry into perioperative small animal fatalities (CEPSAF)

Design: Prospective nested case-control. Multicenter: 117 GP and referrals. United Kingdom. Jun 2002−2004.

Definition: "Perioperative death (including euthanasia) within 48 h of termination of the procedure, except where death was due solely to the surgical or pre-existing medical condition, such that anaesthesia or sedation could not be reasonably excluded as a contributory factor."

Results: 79,178 cat anesthesias. Death risk: 0.24% (*n* = 189).

RF: Poor health status, increasing age, extreme weight, increasing procedural urgency and complexity, endotracheal intubation, fluid therapy. AU: Reported in Brodbelt[19]. COD: reported in Brodbelt.[19]

Brodbelt et al.[19]: The risk of death: the confidential inquiry into perioperative small animal fatalities

Design: Prospective cohort w/ nested case-control. Multicenter: 117 GP and referrals. Jun 2002−2004.

Definition: All animals anesthetized or sedated where there was perioperative death (or euthanasia) within 48 h except where death was due solely to the surgical or preexisting medical condition.

Results: 79,178 cat anesthesias/sedations. Death risk: 0.24% (*n* = 189). Death risk in healthy: 0.11% (81/72,473). Death risk in sick: 1.4% (94/6,705).

RF: Health status, cats increased risk over dogs, post-operative period most common time for death. AU: 10% performed (details not reported). COD: 93% reported (175/189). Deaths classified by an independent review panel of veterinary anesthetists and surgeons based on review of the cases and using a specified list of criteria.

McMillan and Darcy[78]: Adverse event surveillance in small animal anaesthesia: an intervention-based, voluntary reporting audit

Design: Prospective, voluntary reporting. Single center: teaching. United Kingdom. Jun 2012−Jun 2013.

Definition: Voluntary reporting of adverse events over 1 y. No clear definition however they appear to designate intra-anesthetic deaths as death during the anesthesia. The definition/cause of euthanasia is not described.

Results: Total anesthesias: 1,386. Total death risk: 0.43% (*n* = 6). Euthanasia: 5 cases. The study does not distinguish between amount of deaths/euthanasia in dogs and cats. 387 cat anesthesias. Overall compliance: 80.4%.

RF: Cats were more likely to have adverse events than dogs. AU: Not reported. Cause of death: Not reported.

Levy et al.[68]: Perioperative mortality in cats and dogs undergoing spay or castration at a high-volume clinic

Design: Retrospective. Single center: spay-neuter. United-States. 2010−2016.

Definition: “Death occurring in the 24 h period starting with the administration of the first sedation or anesthetic drugs.” Cats and dogs undergoing spay-neuter surgery.

Results: 56,075 cat anesthesias. Death risk: 0.06% (*n* = 34).

RF: Higher risk in females than males. higher risk in cats than dogs. post-operative period when most deaths occurred. AU: Not reported. COD: 82% (*n* = 28) euthanized due to poor anesthetic recovery.

Matthews et al.[74]: Factors associated with anesthetic-related death in dogs and cats in primary care veterinary hospitals

Design: Retrospective matched case-control. Multicenter: 822 GP. United States. 2010−2013.

Definition: Banfield hospitals where death or euthanasia within 7 d of an anesthetic episode for which anesthesia or sedation could not be reasonably ruled out as contributing to death.

Results: 377,565 cat anesthesias. Death risk: 0.11% (*n* = 424).

RF: Cats more at risk than dogs, increasing age, non-elective procedures, when pulse oximetry was not measured, increasing body weight, post-operative period most common for death.

AU: Not reported. COD: Not reported.

Kreisler et al.[64]: Outcomes of elective gonadectomy procedures performed on dogs and cats by veterinary students and shelter veterinarians in a shelter environment

Design: Retrospective, cohort. Single center: spay-neuter. United States. June 2012−Sept 2014.

Definition: Compared the complication rates between veterinary students (VS) and shelter employed veterinarian (SEV) performing spay/neuter procedures on dogs and cats from anesthetic induction to 72 h post-procedure.

Results: 7,699 cat anesthesias (VS: 2,151 cats; SEV: 5,548 cats). Death risk in cats: 0.286% (*n* = 22). Death risk in cats VS: 0.186% (*n* = 4/2,151). Death risk in cats SEVL: 0.324% (*n* = 18/5,548). Total anesthesia (dog and cat): 10,073 total anesthesias. Total death risk: 0.238% (*n* = 24). Death risk VS: 0.13% (*n* = 4/3,048). Death risk SEV: 0.285% (*n* = 20/7,025).

RF: Not reported. AU: 4.5% (1/22). COD: 100% reported (22/22).

Morrison et al.[82]: Development and implementation of veterinary anesthesia medical quality standards for primary care

Design: Qualitative observation with clinical auditing. Multicenter: unclear on total number but there are approximately 1,000 Banfield hospitals, GP. United. Dates unclear Nov 2017−Jan 2020.

Definition: "…any death that occurred within 7 d of a documented general anesthetic procedure." Risk was reassessed after implementing medical quality standards through clinical auditing.

Results: Nov 2017: 12,437 anesthesias. Death risk: 0.096% (*n* = 12). Jan 2020: 14,861 anesthesias. Death risk: 0.182% (*n* = 27). Overall: 350,410 anesthesias. Overall death risk: 0.13% (*n* = 454).

RF: Not reported. AU: Not reported. COD: Not reported.

Varkoulis et al.[117]: A retrospective study on canine and feline mortality during anaesthesia at a university clinic in Greece

Design: Retrospective cohort. Single center: teaching. Greece. Jan 2018−Dec 2019.

Definition: "any death that occurred during anaesthesia, i.e., between induction of general anesthesia with an injectable or volatile anaesthetic agent and cessation of administration of these agents." Animals receiving sedation were also included. Exclusion criteria: insufficient data, euthanasia, or if the animal had >1 anesthesia

Results: 250 cat anesthesias. Death risk 0.8% (*n* = 2) (cardiac arrest 2.8%). ASA classification I or II 0.5%; ASA classification III–V 2.2%.

RF: ASA classification, anesthetic protocol, use of inotropes. AU: Not reported. COD: Not reported.

Brown and Murison[24]: Perioperative anaesthetic complications in healthy cats undergoing anesthesia for neutering in first opinion practice

Design: Retrospective anesthetic record analysis. Multicenter: 3 GP. United Kingdom. Dec 2017−Feb 2021.

Definition: Cats anesthetized for neutering; this included procedures performed under sedation alone.

Results: 1019 cat anesthesias. Death risk 0.1% (*n* = 1).

RF: Did not look at risk factors associated with death. AU: Not reported. COD: Not reported.

Redondo et al.[97]: Anaesthetic mortality in cats: A worldwide analysis and risk assessment

Design: Observational prospective cohort. Multicenter: 198, GP, referral and teaching. Spain, Argentina, France, United Kingdom, United States, Chile, Portugal and Australia. Feb 2016−Dec 2022.

Definition: Cats undergoing anesthesia where death was between preanesthetic medication and 48 h extubation. Deaths were solely associated with anesthesia excluding those related to euthanasia due to preexisting lesions and deaths due to medical/surgery reasons.

Results: 14,962 cat anesthesias. Death risk 0.63% (*n* = 94).

RF: cachexia, higher ASA classification, procedure type (abdominal, orthopedic/neurosurgical, thoracic), mechanical ventilation. AU: Not reported. COD: 94 deaths associated with anesthesia, 21 due to surgery, medical reasons or preexisting injuries, 98 euthanized due to poor prognosis, did not report how these were determined.

Shin et al.[109]: Tiletamine-zolazepam, ketamine, and xylazine anesthetic protocol for high-quality, high-volume spay and neuter of free-roaming cats in Seoul, Korea

Design: Observational, prospective. Single center: Spay-neuter. Korea. 2017−2022.

Definition: All feral cats that underwent sterilization at the clinics. Males were monitored for 24 h and females 72 h.

Results: 1,261 cat anesthesias. Death risk 0.3% (*n* = 4).

RF: Not reported. AU: Not reported. COD: Not reported.

Baker et al.[11]: Risk factors affecting all-cause mortality in cats hospitalized by a referral soft tissue service

Design: Observation, retrospective. Single center: teaching. United States. 2015−2020.

Definition: All-cause mortality rate in cats hospitalized by the soft tissue surgery service.

Results: 492 cat anesthesias. Death risk: all-cause mortality 6.1% (*n* = 30). 7 deaths, 23 euthanasias.

RF: Higher ASA classification, emergency surgery. AU: Not reported. COD: euthanasia was elected in cases with poor prognosis or finances and poor prognosis. 3 deaths during surgery (2 cardiopulmonary arrest, 1 massive hemorrhage). 4 deaths after surgery (1 respiratory compromise, 3 cardiopulmonary arrest).

ASA = American Society of Anesthesiologists physical status classification; AU = autopsies, if reported, the percentage of deaths that had an autopsy performed; COD = cause of death = the percentage of all deaths in which a cause of death was reported; Death in healthy = percentage of deaths in ASA I or II animals; Death in sick = percentage of deaths in ASA III–V animals; Death risk = total number of deaths out of all anesthetic procedures; Design = study design and the number and types of centers included; Definition = definition of perianesthetic death, including length of follow-up and any inclusion or exclusion criteria, if reported; DIC = disseminated intravascular coagulation; GP = general practice; Referral = specialty practices; Results = total number of anesthetic procedures. Questionnaire response rates (percentage of number returned out of total). RF = major risk factors associated with perianesthetic death; RR = response rate; Spay-neuter = facilities performing only de-sexing procedures; teaching = university teaching hospital.
